# ARYANA: Aligning Reads by Yet Another Approach

**DOI:** 10.1186/1471-2105-15-S9-S12

**Published:** 2014-09-10

**Authors:** Milad Gholami, Aryan Arbabi, Ali Sharifi-Zarchi, Hamidreza Chitsaz, Mehdi Sadeghi

**Affiliations:** 1 Department of Computer Science, University of Maryland, College Park, MD, USA; 2 Department of Computer Science, University of Toronto, Toronto, ON, Canada; 3 Institute of Biophysics &. Biochemistry, University of Tehran, Tehran, Iran; 4 Stem Cells and Developmental Biology Group of Cell Science Research Center, Royan Institute for Stem Cell Biology and Technology, ACECR, Tehran, Iran; 5 Department of Computer Science, Colorado State University, Fort Collins, CO, USA; 6 National Institute of Genetic Engineering and Biotechnology, Tehran, Iran

**Keywords:** Alignment, Read mapping, DNA sequencing, Next generation sequencing (NGS)

## Abstract

**Motivation:**

Although there are many different algorithms and software tools for aligning sequencing reads, fast gapped sequence search is far from solved. Strong interest in fast alignment is best reflected in the $10^6 ^prize for the Innocentive competition on aligning a collection of reads to a given database of reference genomes. In addition, *de novo *assembly of next-generation sequencing long reads requires fast overlap-layout-concensus algorithms which depend on fast and accurate alignment.

**Contribution:**

We introduce ARYANA, a fast gapped read aligner, developed on the base of BWA indexing infrastructure with a completely new alignment engine that makes it significantly faster than three other aligners: Bowtie2, BWA and SeqAlto, with comparable generality and accuracy. Instead of the time-consuming backtracking procedures for handling mismatches, ARYANA comes with the seed-and-extend algorithmic framework and a significantly improved efficiency by integrating novel algorithmic techniques including dynamic seed selection, bidirectional seed extension, reset-free hash tables, and gap-filling dynamic programming. As the read length increases ARYANA's superiority in terms of speed and alignment rate becomes more evident. This is in perfect harmony with the read length trend as the sequencing technologies evolve. The algorithmic platform of ARYANA makes it easy to develop mission-specific aligners for other applications using ARYANA engine.

**Availability:**

ARYANA with complete source code can be obtained from http://github.com/aryana-aligner

## Introduction

Every living cell carries a *book of life *consisting of several thousand to billions of characters with answers to many vital questions. Human efforts to decipher that book has gained increasing momentum since 1953 when the double helical structure of DNA was discovered. Twenty years later. W. Gilbert and A. Maxarn read the first 24-character word of the book [1]. when F. Sanger and his colleagues were developing another sequencing method based on the application of labeled dideoxynucleotide triphosphates that act as chain-terminators in a PCR reaction [2,3].

About three decades after the first DNA sequencing, the dream of reading the human book of life was realized by completion of the *human genome project *[4-6]. The *International Human Genome Sequencing Consortium *used a laborious hierarchical process to divide the genome into smaller covering tiles while the *Celera Genomics *firm replaced that by a computational sequence-assembly software applied to the data generated from blindly shredded (shotgun) whole genome [7,8]. The automated Sanger method was the gold standard for about two decades, as the *first generation *of DNA sequencing, until increasing demand for fast and inexpensive methods to produce high volume of error-free genomic information caused emergence of new technologies, the so called *Next-Generation Sequencing (NGS) *[9].

A paradigm shift in both the experimental techniques and computational methods occurred due to the transition to the NGS technologies and also availability of finished reference genomes, such as the human genome, for more than 2000 prokaryotes. eukaryotes and Archaea. Long, accurate, expensive Sanger mate-paired reads (~ 400 to 750 bp) [11] which were mostly used for *de novo *sequencing and assembly are now replaced by several fold more (ultra-)short. erroneous, but inexpensive NGS reads. There is significant ongoing effort for the *de novo *assembly [11] of NGS data in combination with additional information such as long reads and optical maps [12] in order to uncover the whole genomes of different organisms. However, the vast majority of NGS data generated today in transcriptomics. epigenomics. and variation studies belong to the organisms with identified whole-genomes. which are mapped to the existing reference genomes using short or long read aligners. Emergence of the 1000 human genome project to catalogue all of the human genome variants through population resequencing is a good representative evidence for this paradigm shift [13].

In the new paradigm, aligning reads to a reference sequence lies in the core of numerous different applications including detection and annotation of single nucleotide polymorphisms (SNPs) [14-17], structural and copy number variations (CNVs) [18,19], detection and alignment of transcript variants and splicing [20-22], and browsing and visualization [23-26]. There is a wide range of software available to process the NGS data from lightweight tools working on a small desktop [25] to more sophisticated resources designed for clouds [27-29].

Although there are many different algorithms and software tools for aligning NGS reads [30-41], of which BWA [42,43] and Bowtie [44,45] have been extensively used in many studies mainly due to their low memory footprint and fast and highly accurate results, fast gapped sequence search is still far from solved. A good evidence is the 10^6 ^prize of the Innocentive competition [46] entitled "Identify Organisms from a Stream of DNA Sequences" on aligning a collection of NGS reads, generated by diverse platforms including Illumina, Roche 454, Ion Torrent, and Pacific Biosciences, to a given database of reference genomes.

Here we introduce our seed-and-extend aligner called ARYANA which is a fast and general purpose solution with on-par accuracy and small memory usage. We compare ARYANA with other aligners: Bowtie2 [45], BWA-SW [43], and SeqAlto [30]. ARYANA is multiple times faster than all of these aligners with comparable generality and accuracy. This superiority in performance is revealed more as the read length increases, which is in perfect harmony with the fact that the read length is increasing as the NGS technologies evolve.

## Methods

Every read is individually aligned by ARYANA, which enables using it in distributed computing frameworks by partitioning the input read data set, in addition to the multithreaded parallel infrastructure embedded in ARYANA that permits complete CPU usage when running on a multi-core machine.

Alignment of a single read consists of two main phases:

• In the first phase of the algorithm ARYANA extracts a set of seeds from the read sequence that satisfies certain conditions. These conditions and the approach for extracting these seeds are explained in the sections searching for the exact matches of a seed and seed extraction. For each exact match of these seeds in the reference genome. ARYANA grants score to some corresponding genomic region. The genomic regions are represented by partitions of the reference genome called *tags*. The scores provide a preliminary criterion for ranking the tags based on their associated genomic region's similarities to the read. Details of how the tags are defined and handled and the scoring system is explained in sections tags and scoring and accessing and updating tag information.

• In the second phase we focus on the tags that received the highest scores during the first phase and consider them as candidates for the final alignment. The read is more precisely aligned to each of these candidate regions by using a differential-position dynamic programming algorithm to find the region which has the best alignment. More details of the second phase of the algorithm is available in section precise alignment to the candidate segments.

Searching for the exact matches of a seed

ARYANA uses the Burrows-Wheeler Aligner (BWA) implementation of the Burrows-Wheeler transform (BWT) and Ferragina-Manzini index (FM-index) [43,47] to search for exact matches of a seed. To ensure the reverse DNA strand is also being considered, the reverse complement of the reference genome is attached to the end of the forward genome, and index tables are constructed for the double sized reference.

We define two search procedures that work by using this data structure:

• *forward exact search: *The search process is performed in several iterations, starting from the rightmost letter of the seed and extending the suffix one letter per iteration to the left. At each iteration we have access (with *O*(1) time complexity) to list of the exact matches of the current suffix.

• *backward exact search*: Since the index tables are built by concatenation of the reference genome to its reverse complement, we can search in the opposite direction, from left of the seed to the right, by performing forward exact search on the reversed complement of the seed. Although the matches found by this search are reverse complements of the original seed, we can still find out how far we can continue matching and extending the prefix of the original seed (which corresponds to the suffix for the reversed complement of the seed).

By using this data structure we can find list of the BWT indices for all matches of a suffix in *O*(*k*) where *k *is length of the suffix. We should note that finding the genomic positions from BWT indices can be done relatively fast. Furthermore, our experiments showed ARYANA consumes about 5.1 GB of memory when aligning reads to a human genome which is an amount that even today's typical personal computers can provide.

### Seed extraction

#### Which seeds to extract?

For aligning each read ARYANA extracts a maximal set of seeds that has the following conditions:

1 Each seed has at least *k *basr pairs.

2 No couple of seeds overlap more than *k *basr pairs.

3 Each seed has at least one exact match in the reference genome.

4 The seeds are maximal: i.e. if we extend a seed the set no longer remains valid.

The value of k is decided dynamically by ARYANA, being 16 for reads shorter than 50 bp and increased for longer reads.

There are three main reasons for having these conditions. Firstly, we force the seeds to have some minimum size and to be maximal in order to avoid the seeds that have too many matches in the reference. These seeds generally do not help distinguishing the correct region among its rivals. Secondly, it is possible for a seed to not match to the correct region due to some error or variant but to match to another region. In this case we do not want to lose all other seeds that overlap with this seed. This is why we have allowed overlaps with less than *k *base pairs. Thirdly, by limiting the size of the overlaps the total number of seeds and their lengths reduces thus the speed improves.

#### How to extract these seeds?

**Figure 1:**
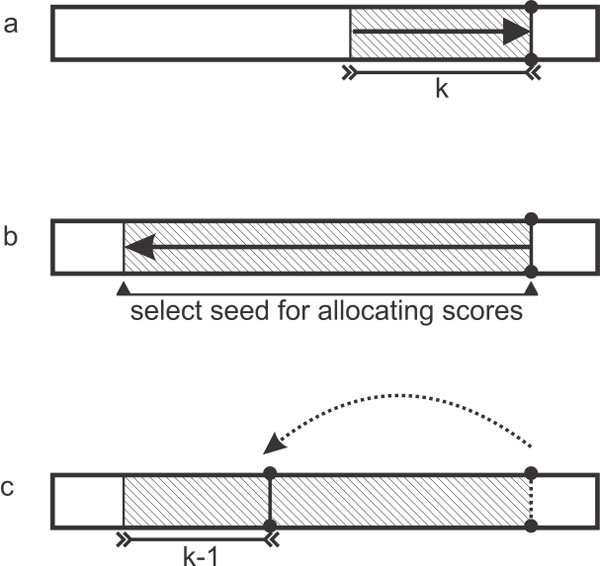
**Maximally extending a seed exact match, (a) When there is an exact match of length k for the seed sequence, (b) it is extended to the left as much as possible and the extended seed is granted score**. Afterwards, (c) the *k *− 1 leftmost letters from the seed are kept for the next iteration of MAXIMALLYSEED in Algorithm 1.

ARYANA starts from the right end of the read and uses a greedy iterative approach for extracting the valid set of seeds. At the beginning of each iteration ARYANA considers an initial seed that is the rightmost substring of length *k *which is not fully covered by previously extracted seeds. Next the algorithm starts matching this seed to the reference, with the direction from left to right, by using reverse exact search described in section searching for the exact matches of a seed. If it successfully matched the whole seed, it then extends the seed maximally towards left by using forward exact search described in section searching for the exact matches of a seed. This maximal seed is then extracted to use its found matches in the reference for scoring the related tags (Figure 1).

**Figure 2:**
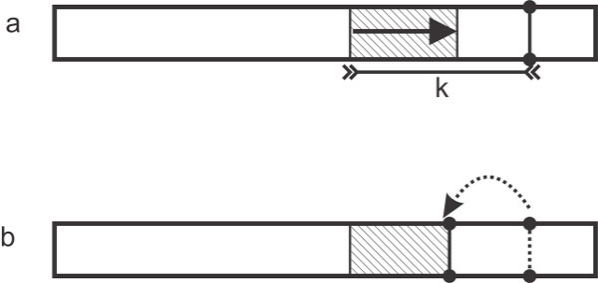
**No exact match of the seed exists, (a) When the seed sequence of length k has no exact match in the reference genome, (b) only those leftmost letters in the seed that have a short exact match are kept, and the right tail of the seed is trimmed for the next iteration of MAXIMALLYSEED in Algorithm 1**.

If the reverse exact search fails to match the whole seed, assume it fails when trying to match the *j*th base pair (*j *<*k*), every seed that contains this substring of length *j *will also fail to find a match in the reference. To reduce the work for the next iterations we can jump over this *j *base pairs by behaving as the *k *− *j *base pairs left to this *j*-length substring was covered by previously extracted seeds (Figure 2).

Algorithm 1 is a pseudo code for the above procedure. MATCHLEFTTORIGHT(*seq, s, max*) is based on the reverse exact match introduced in section searching for the exact matches of a seed. It matches at most match of *seq *to the reference starting at *s*, moving from left to right. It returns the length of the matched string. MATCHLEFTTORIGHT(*seq, s, max*), which is based on the forward exact match introduced in section searching for the exact matches of a seed, does the same except moving from right to left. It returns BWT indices for the beginning and end of the matched region and length of the matched string. BWTPOSITION(*index*) returns the reference position of *index*, where *index *is a BWT index. GRANTSCORE(*pos, s*) grants score for the tag associated with the position *pos*, and adds *s *points to its score. The scoring system and the tags are explained in section tags and scoring.

**Algorithm 1 **extracting seeds

   **function **MAXIMALLYSEED(*seq, k*)

    *right ← *LENGTH(*seq*)

    **while ***right *≥ *k ***do**

      *matched *← MATCHLEFTTORIGHT(*seq, right − k *+ 1, *k*)

      **if ***matched *<*k ***then**

        *right *← *right *− *k + matched*

        **continue**

    **end if**

    *begin, end, matched *← MATCHRIGHTTOLEFT(*seq, right*, INF)

    **for ***index *from *begin *to *end ***do**

      *pos *← BWTPOSITION(*index*)

      GRANTSCORE(*pos* − (*right − matched *+ 1), *matched*)

    **end for**

    *right *← *right* − *matched + k *− 1

  **end while**

end function

### Tags and scoring

**Figure 3:**
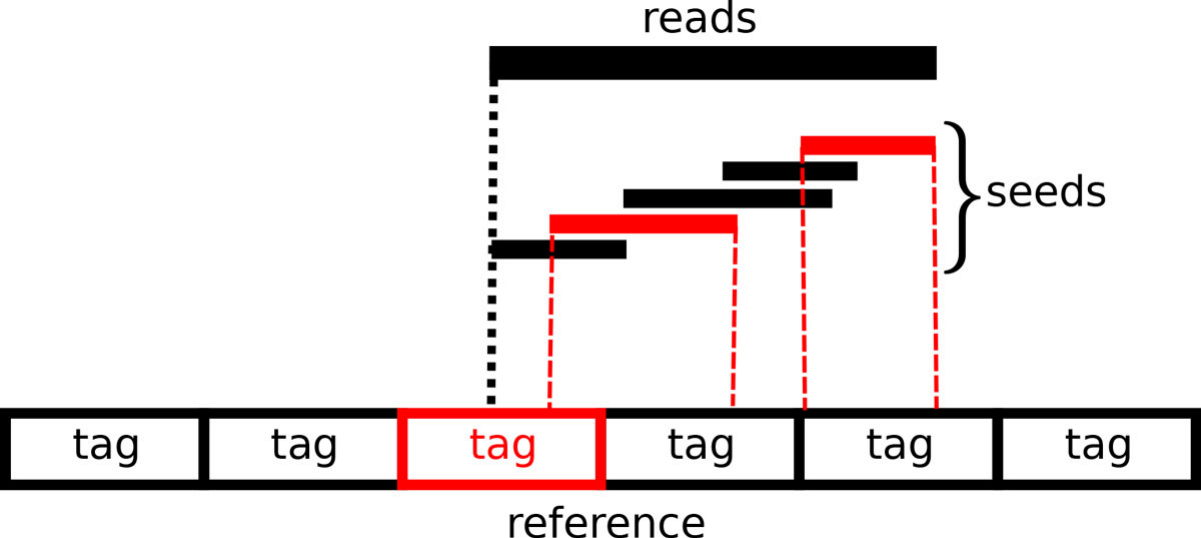
**Scoring the tags according to the seeds**. The red seeds are the ones which have exact matches in this region of the reference. These seeds influence the score of the red tag which includes the relative start position of the whole read.

We define *tags *as consecutive non-overlapped partitions of the reference genome, each of length *L*. In our implementation we have $L=\frac{R}{c}$ where *c *is a constant integer (with the default value 10) and *R *is the length of the read. The intuition is that each tag is an approximate region around the leftmost position of the inexact match of the whole read sequence. Tags are not defined to cover the whole match of a read and only the start position of the read has to be inside the tag. In Figure 3. a read sequence with some matched seeds and the corresponding tag are depicted.

Each exact match of a seed increases the score associated with exactly one of the tags, which is the one that contains the start position of the whole read. To find this tag we compute the relative start position of the read and update score of the tag containing this position (Figure 3). More precisely, if there are *n *letters before the seed in the read, and the match position starts at the *m*-th letter of the genome we estimate *m *− *n *to be roughly the start position of the read if it were to be aligned to the genome accordingly. This way the consecutive seeds of the same read will produce similar estimated read start positions if their exact match locations are consecutive.

The actual start position of the read might be slightly different from the estimated value due to possible indels. Likewise, the start positions estimated for consecutive matches of different seeds of the same read might slightly differ: however, the estimated start positions fall into one or at most two adjacent tags if the total size of indels inside the read is less than *L*.

For each exact match of the seed, the tag containing estimated start position of the read is granted a score equal to the length of that seed. As a result the final score of each tag will be sum of the size of the seeds that correspond to this tag. In case of too small seed lengths or repeat elements where there might be many exact matches for the same seed sequence, only the first *P *matches are granted the scores, where the default value for *P *is 50 but can be changed through command-line parameters.

Because of using non-overlapped tags there is the possibility of dividing the total score regarding one match of the read between two adjacent tags. This happens in extreme cases where the read's start or end position is near the boundaries of a tag and at the same time there is an indel inside the read: however this is not a significant problem as we consider several candidate tags for the second phase.

### Accessing and updating tag information

There are a total of *G*/*L *tags, where *G *and *L *are the lengths of the genome and the tags, respectively. A simple way is to assign tag scores to an array of size *G*/*L*, which might not seem a problem at the first glance. However, it takes long time to reset the whole array for each read, and the storage space would also be considerable if there are multiple threads aligning the reads simultaneously. To address this challenge, ARYANA keeps track of only those tags that have a non-zero score in a hash table with open-addressing collision management that provides fast access to the records. Upon granting some score to a tag, first the segment number is looked up in the hash table and if found its score is updated: otherwise, a new record is assigned and inserted into the hash table.

While the hash table size is considerably smaller than the total number of tags, it still takes considerable time to free it upon a new read. Additionally, each hash record contains the read ID for which the scores were granted. While looking up a tag, all hash records belonging to the previous reads are ignored and the corresponding cells of hash table are treated as if empty. Hence, there is no need to reset the hash table on a new read, which has a great impact on efficiency of ARYANA. Furthermore, to get rid of scanning the hash table for selecting the top score tags, a dynamic list keeps track of the *t *top-scoring tags, where *t *is 10 by default. The list is updated if necessary following each update in the hash table.

In addition to the tag number as the hash key and the tag score, for each tag we store the seed information for all of the seeds that have resulted in updating its score. This information includes the seed length, its position in the read sequence and the genomic position of its match in reference.

### Precise alignment to the candidate segments

The *t *top scoring tags are selected for the second phase of the algorithm that performs a precise dynamic programming alignment of the given read to each of the regions associated with the candidate tags and finds the best overall alignment consisting of matches, mismatches and indels (the so-called CIGAR sequence in the SAM files). The region associated to the tag is extended e nucleotides (20 bp by default) from both sides to ensure the potential alignment region of the read is completely covered by the extended segment.

**Figure 4:**
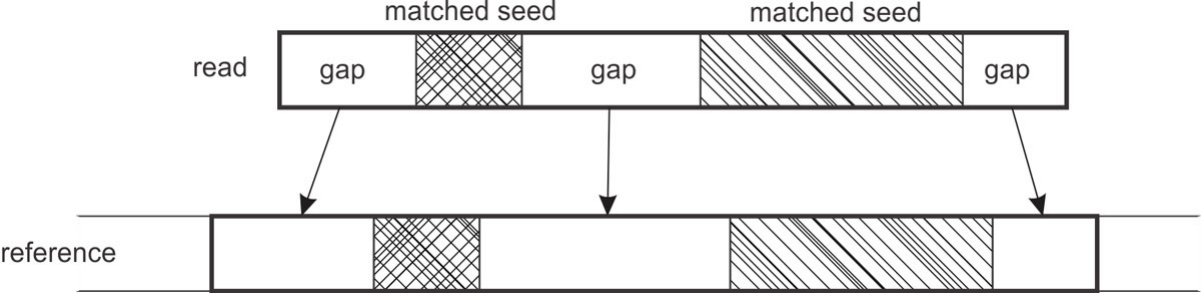
**Gap-filling dynamic programming**. To make a complete alignment between the read and the reference genome, only gaps between matches seeds are aligned using a variant of the Needleman-Wunsch dynamic programming [48].

For the sake of performance we use those seeds of the read that were associated to this tag in the previous phase. A consistent and non-overlapping subset of these matched seeds are selected greedily as a fixed set of matched blocks. The algorithm performs dynamic programming alignment only on the gaps between these blocks (Figure 4).

The dynamic programming algorithm we used for aligning the gaps is a variant of the Needleman-Wunsch algorithm [48] that tries to align two sequences to each other with the minimum number of mismatches and indels (in our implementation mismatches and indels have the same cost). The dynamic programming matrix has two dimensions: (i) position inside the first sequence, and (ii) the difference between the positions inside the second and the first sequence. The dynamic programming matrix is updated as

$$D\left[i\right]\left[\text{offset}\right]=\text{min}\left\{\begin{array}{cc} D\left[i-1\right]\left[\text{offset}\right]+I\left(\text{ref}\left[i+\text{offset}\right]==\text{read}\left[i\right]\right) & \text{Match/Mismatch}\\ D\left[i-1\right]\left[\text{offset + 1}\right]+1 & \text{Insert}\\ D\left[i\right]\left[\text{offset}-1\right]+1 & \text{Delete} \end{array}\right)$$

Above, ref and read are the reference and the read respectively, *I *[true] = 0, *I *[false] = 1, and 0 <*i *< length (*gap*) and |offset| <*d *for the dimension sizes in which length(*gap*) is the size of the gap in the read and *d *is the largest difference between the sizes of any two corresponding subsequences on the best alignment path. This algorithm has the running time of *O*(*dn*) and is faster than the regular Needleman-Wunsch algorithm for a limited *d*.

### Aligning paired-end reads

For paired-end data, ARYANA aligns each read separately and finds a couple of match groups, each containing *t *best matches of one read to the reference (the default value for *t *is 10). It then looks for a pair of matches one from each group that meet the requirements given for the paired alignment, including maximum and minimum distances between the reads and their relative orientation. In the case of multiple answers, the total scores for each pair of matches are used to rank them and report the best pair.

## Results and discussion

We compared ARYANA with three other NGS aligners in terms of speed and accuracy. We selected BWA and Bowtie2 as the two most widely cited aligners and also SeqAlto which is a more recent aligner that outperforms many other recent aligners. All aligners were tested with the default parameters but executed with multithread in some experiments.

The experiments were performed on a platform with 48 AMD Opteron Processor 6174 CPUs each having 12 cores with clock speed of 2.2 GHz. The hg19 human genome assembly was used as the reference for all test cases. We used dwgsirn (https://github.com/nh13/DWGSIM/wiki) to simulate data sets similar to real reads produced by Illumina NGS platforms.

**Figure 5:**
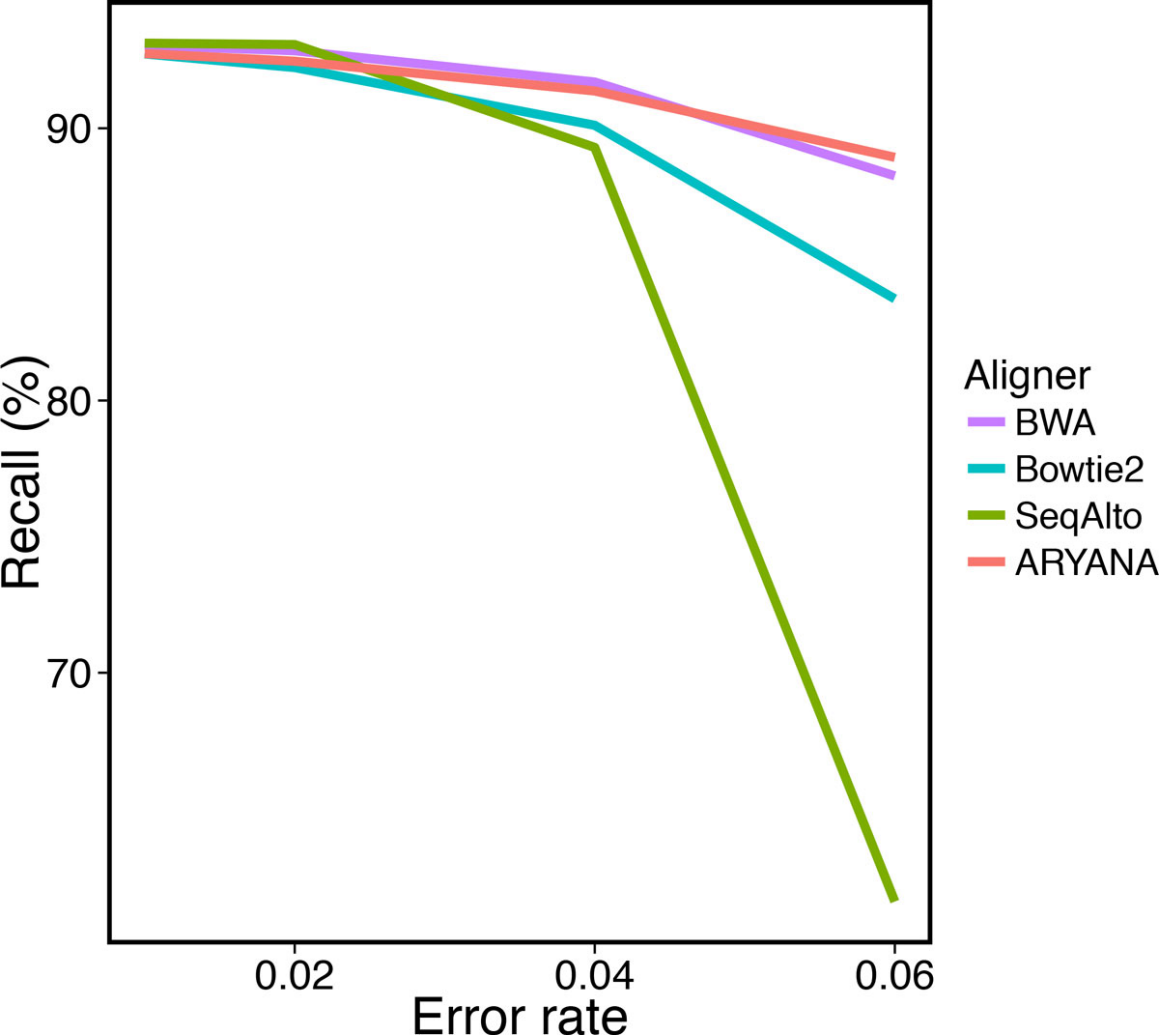
**Recall of the aligners for experiments on data with different error rates**.

**Figure 6:**
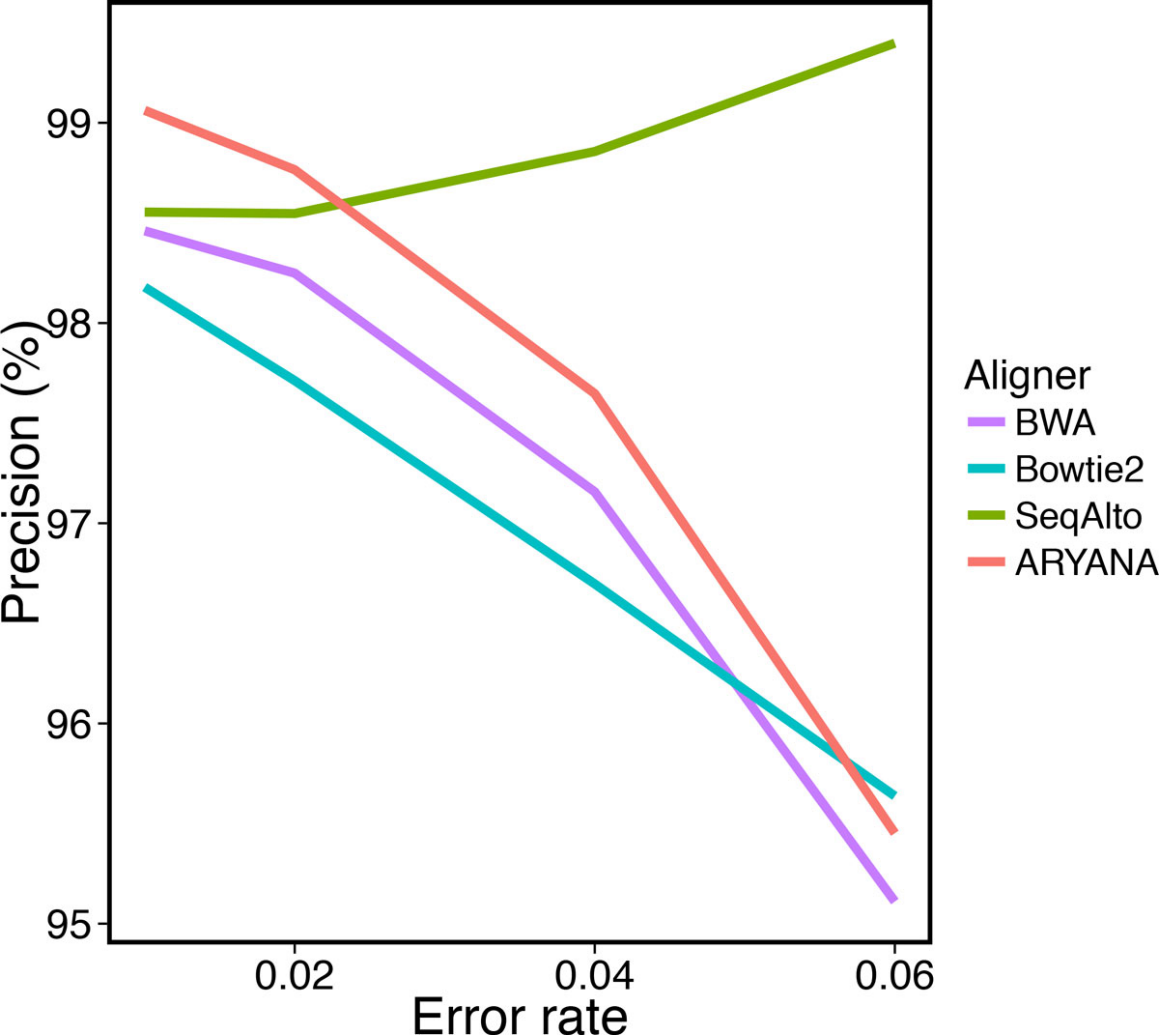
**Precision of the aligners for experiments on data with different error rates**.

**Figure 7:**
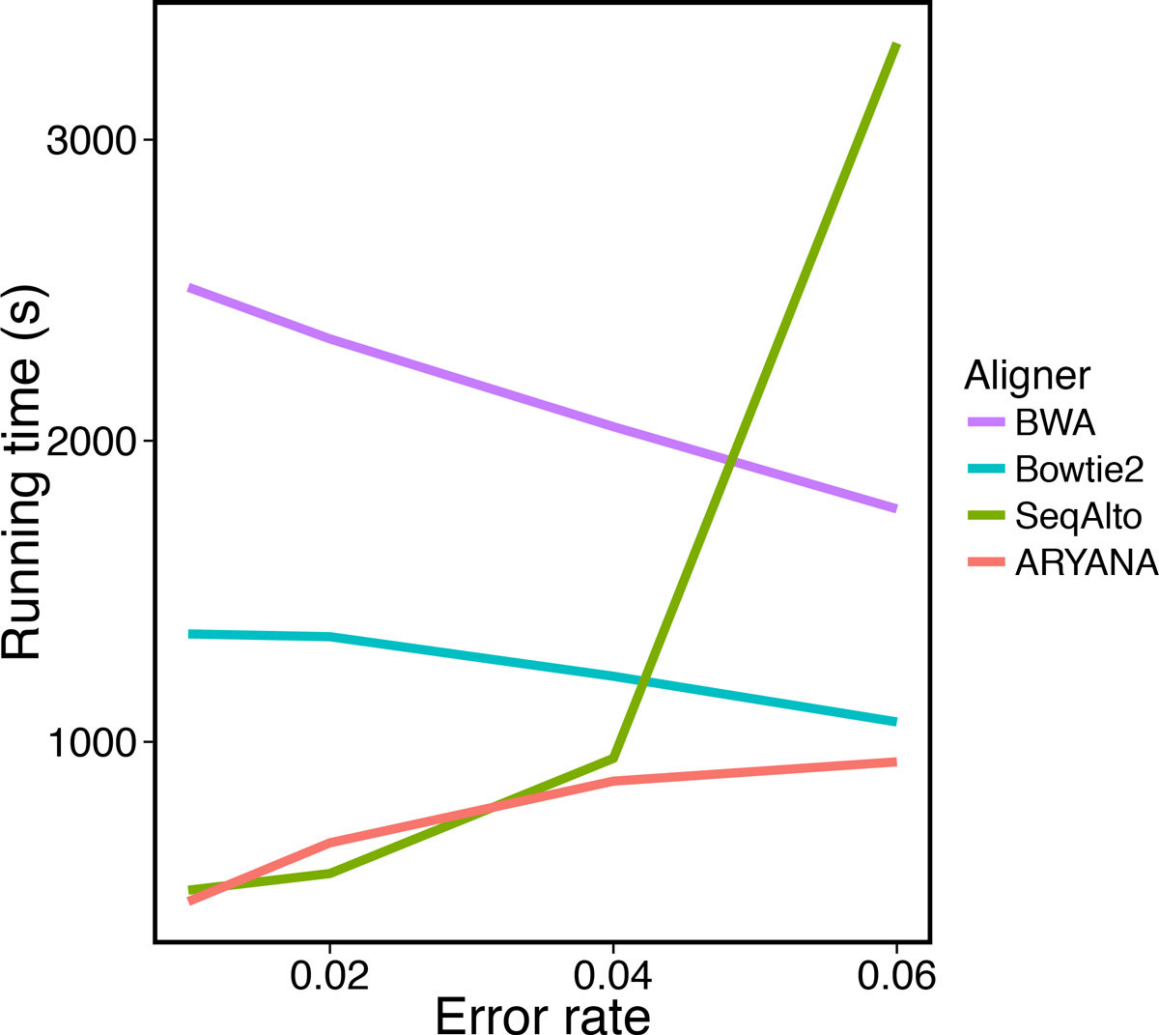
**Running time of the aligners for experiments on data with different error rates**.

To study the behavior of the aligners in response to sequencing errors, we generated four data sets with different error rates, each containing 1 million 200 bp reads. We measured recall, precision and running time of the aligners on these data sets (Table 1). All aligners exhibited almost similar recall when the error rate was up to 2%, but at 4% error rate, ARYANA and BWA outperformed the others, and at 6%, the difference was much more significant (Figure 5). SeqAlto showed higher precision compared to other aligners on reads with high error rate. However this was not surprising as at the same time it demonstrated very low recall on the same reads (around 61.59% for reads with 6% error rate). The same happens for Bowtie2 with a smaller scale on reads with 6% error rate. Other than these, ARYANA showed higher or almost similar precision (Figure 6). The running times of ARYAN A and Seq Alto were relatively similar for reads with low error rates (2% and 4%). but Seq Alto's running time significantly increased for reads with higher error rate (6%). For all error rates ARYANA had less running time than Bowtie2 and BWA (Figure 7).

**Table 1:** Time (s), recall (%), and precision (%) for aligning reads with different error rates.

	1% error			2% error			4% error			6% error		
	**T(s)**	**R(%)**	**P(%)**	**T(s)**	**R(%)**	**P(%)**	**T(s)**	**R(%)**	**P(%)**	**T(s)**	**R(%)**	**P(%)**

ARYANA	469	99.06	92.74	664	98.76	92.46	869	97.64	91.37	933	95.45	88.93

SeqAlto	507	93.12	98.55	562	93.07	98.54	945	89.29	98.85	3320	61.59	99.39

Bowtie2	1358	92.73	98.17	1349	92.23	97.71	1217	90.10	96.69	1066	83.73	95.63

BWA	2509	93.04	98.46	2338	92.84	98.24	2046	91.69	97.15	1773	88.25	95.11

For each test case, a data set including 1 million 200 bp single simulated Illumina reads was processed.

**Figure 8:**
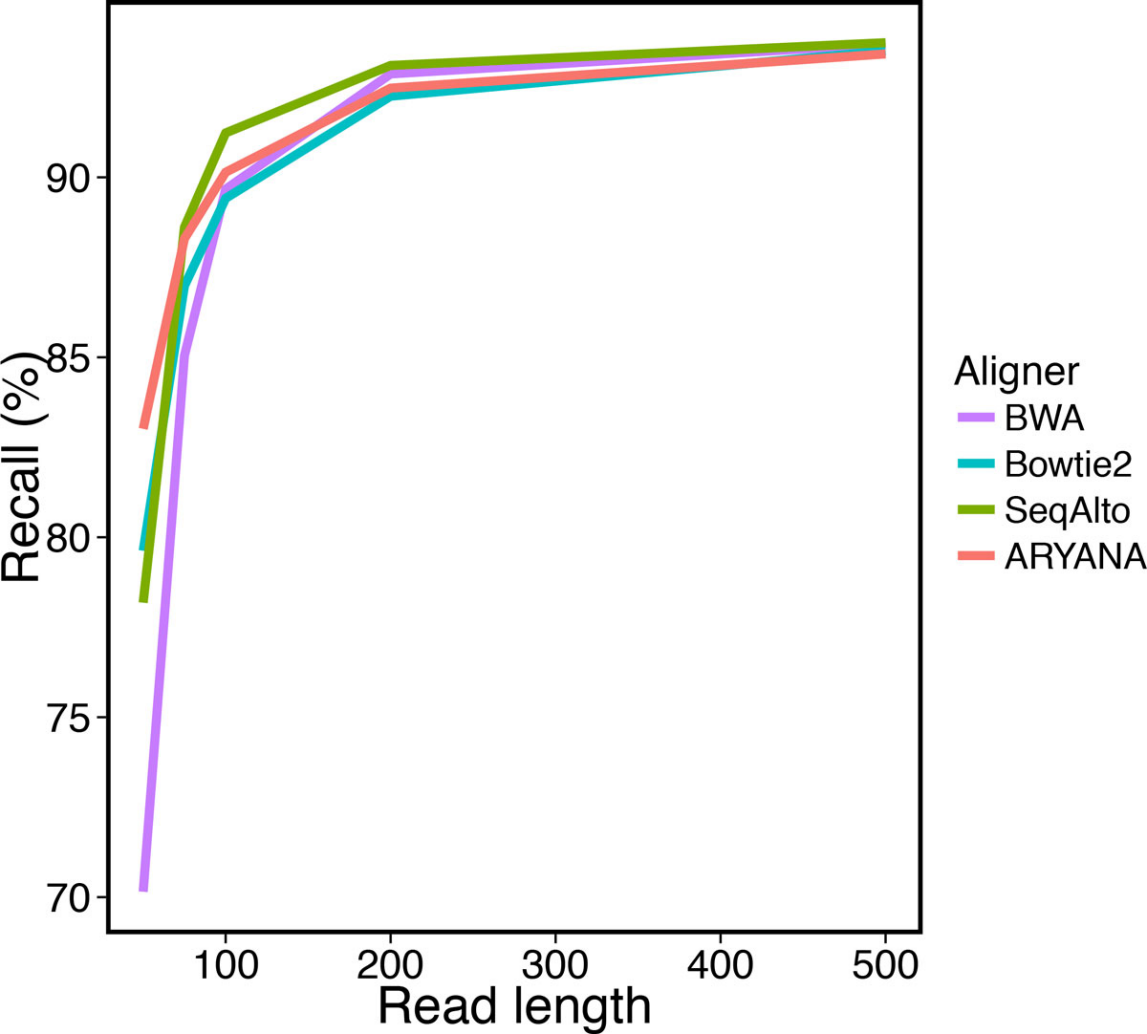
**Recall of the aligners for experiments on data with different read lengths**.

**Figure 9:**
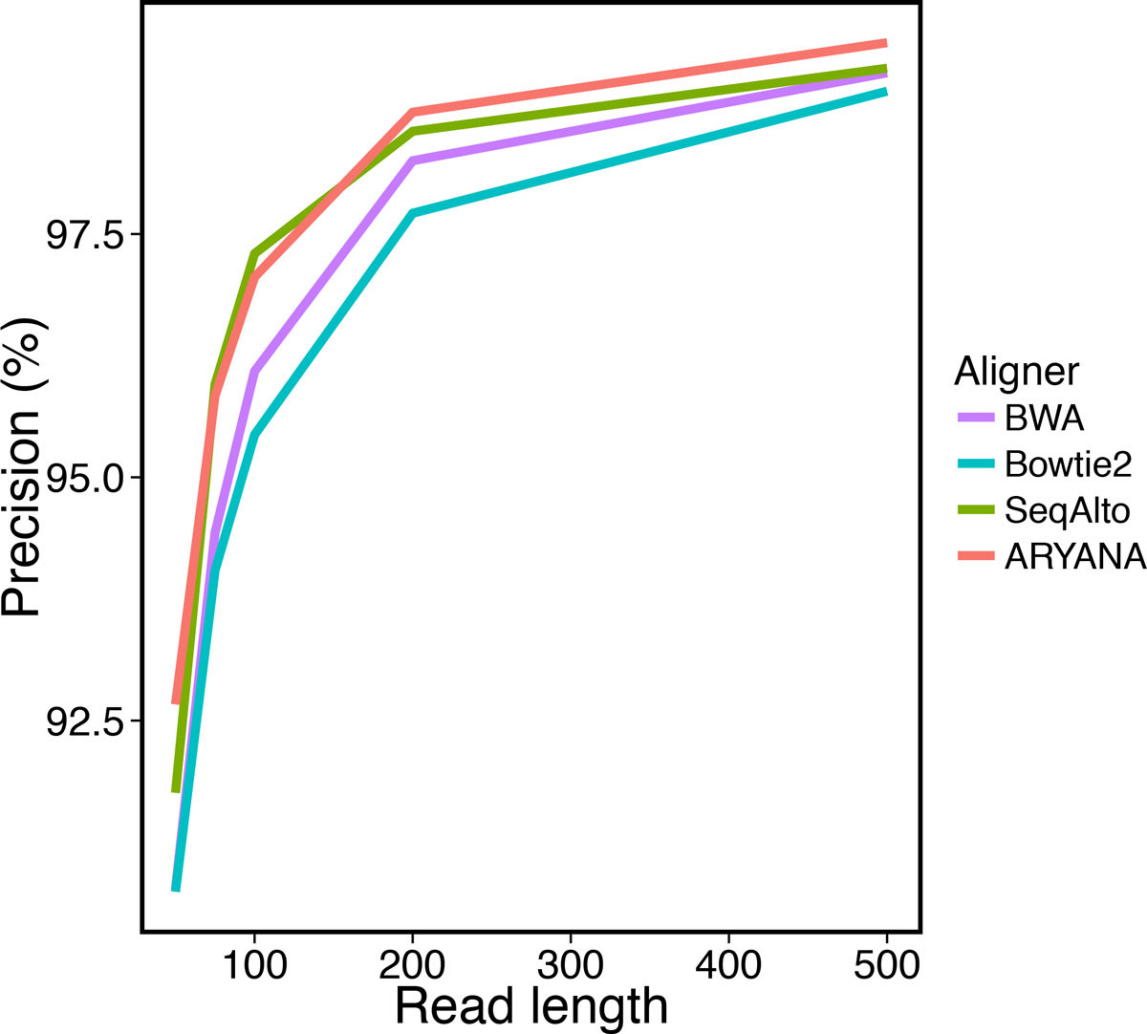
**Precision of the aligners for experiments on data with different read lengths**.

**Figure 10:**
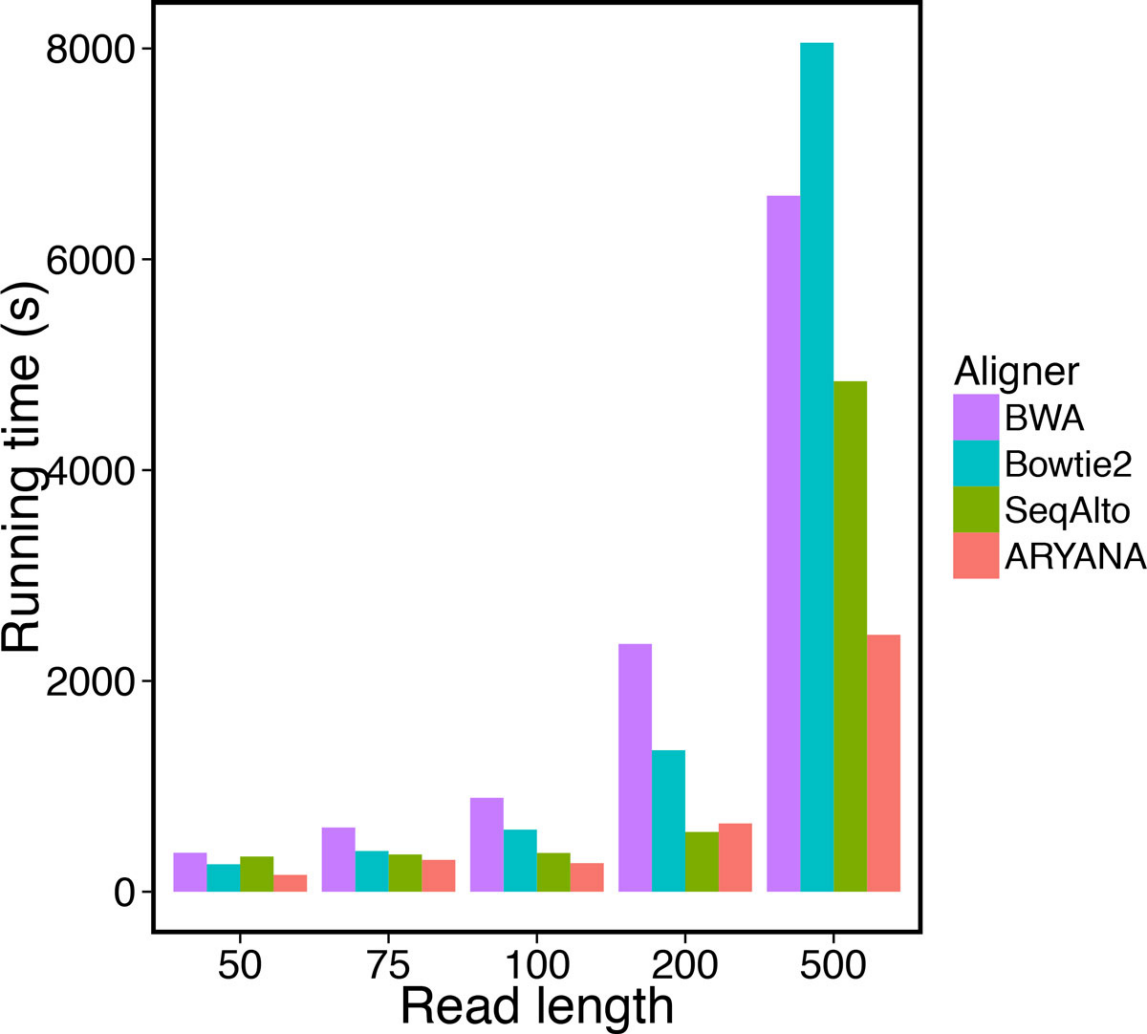
**Running time of the aligners for experiments on data with different read lengths**.

We tested the aligners on different read lengths, from 50 to 500 bps. with a fixed 2% error rate. The data set for each test contained 1 million reads. We measured recall, precision and running time of the aligners on these data sets (Table 2). ARYANA had higher recall than other aligners for short reads (i.e. 50 bp) with 83% recall, while the second best was Bowtie2 with 79% recall (Figure 8). Also it demonstrated superior precision in comparison with the other aligners (Figure 9). As the read lengths increased, the aligners exhibited closer accuracies but divergent running times. ARYANA aligned 500 bp reads almost twice faster than SeqAlto. the second fastest aligner, and more than 3.3 folds faster than Bowtie2. The exponential-like uptrend of the running times shows that ARYANA would be even much faster than the other aligners for longer reads (Figure 10).

**Table 2:** Ti me (s), recall (%), and precision (%) for aligning reads with different read lengths.

	50 bp			75 bp			100 bp			200 bp			500 bp		
	**T(s)**	**R(%)**	**P(%)**	**T(s)**	**R(%)**	**P(%)**	**T(s)**	**R(%)**	**P(%)**	**T(s)**	**R(%)**	**P(%)**	**T(s)**	**R(%)**	**P(%)**

ARYANA	159	83.01	92.66	302	88.29	95.83	271	90.14	97.05	647	92.47	98.75	2437	93.42	99.46

SeqAlto	333	78.18	91.75	353	88.61	95.95	367	91.23	97.29	567	93.10	98.55	4843	93.74	99.20

Bowtie2	260	79.62	90.74	386	86.98	94.04	589	89.41	95.43	1342	92.25	97.71	8054	93.51	98.96

BWA	369	70.15	90.76	609	85.04	94.43	890	89.67	96.08	2350	92.87	98.25	6603	93.70	99.15

For each test case, a data set including 1 million Illumina reads with fixed 2% error rate was processed.

To see how ARYANA works on the real data, we compared the aligners on two datasets SRR946843 (http://trace.ncbi.nlm.nih.gov/Traces/sra/?run=SRR946843) and SRR003161 (http://trace.ncbi.nlm.nih.gov/Traces/sra/?run=SRR003161). The SRR946843 dataset has been generated by the very recent Ion Torrent PGM technology with the average read length of 172 bp. and the SRR003161 dataset is produced by Roche 454 with an average read length of 572 bp as a part of the 1000 human genomes project.

**Figure 11:**
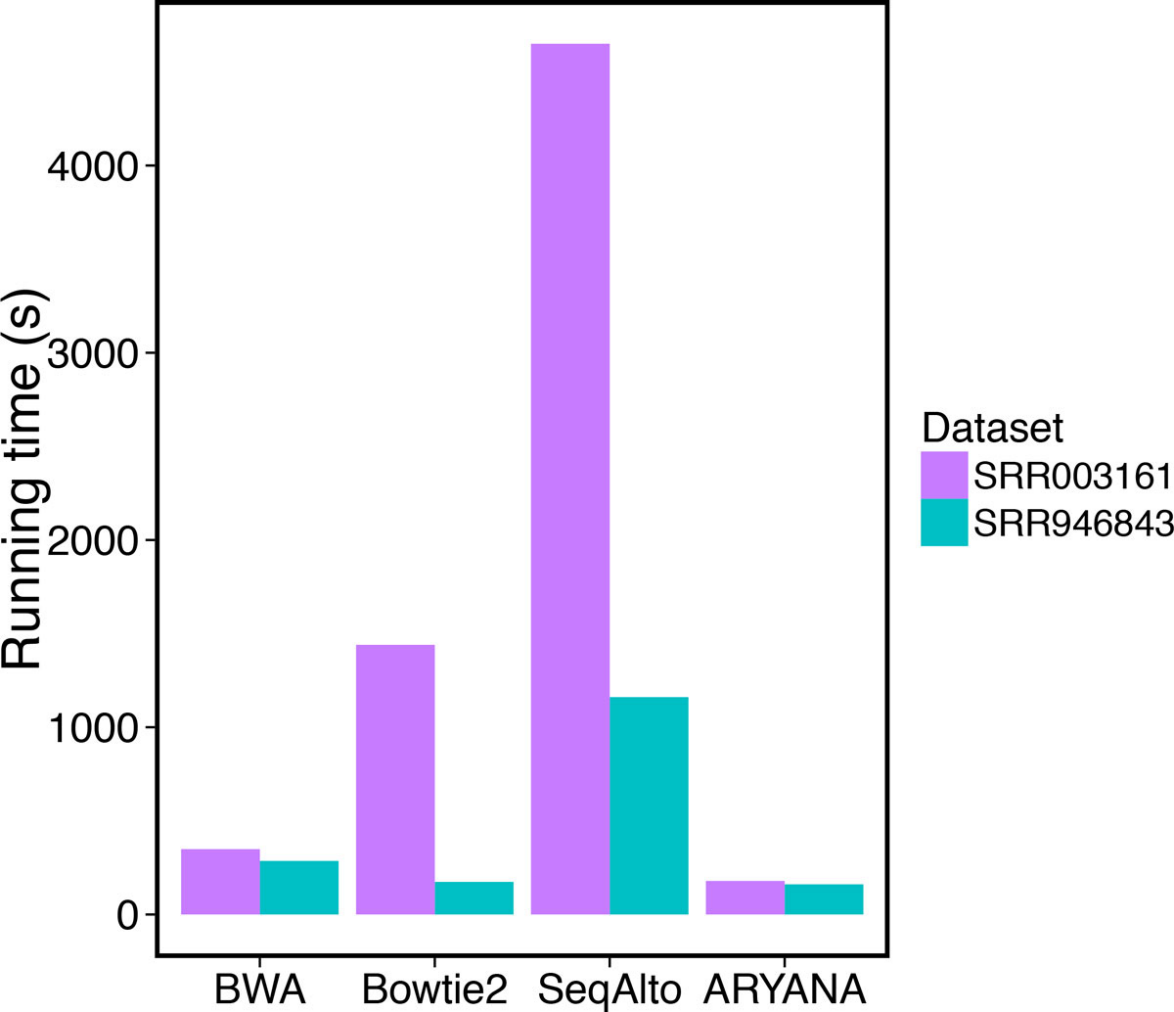
**Running time of the aligners for experiments on SRR946843 and SRR003161 data sets**.

**Figure 12:**
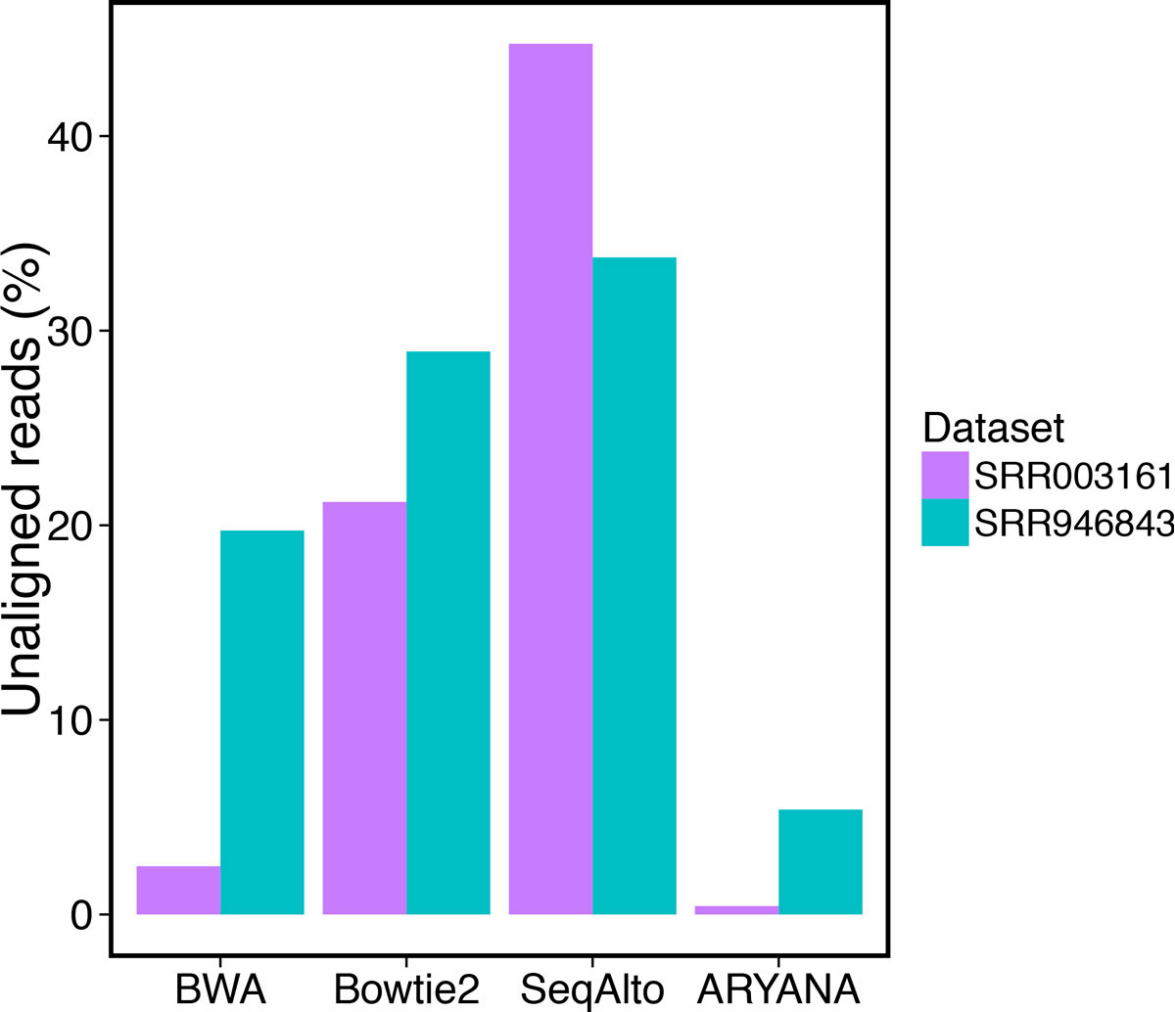
**Number of unaligned reads for experiments on SRR946843 and SRR003161 data sets**.

All the aligners were executed with 48 threads in parallel, on identical cluster machines having 48 AMD Opteron(tm) processors at 2200 MHz speed and 64GB of memory, running Scientific Linux release 6.4 (Carbon). For both data sets. ARYANA was significantly faster than all of the other aligners, particularly on the 454 reads as they are longer in average. SeqAlto was significantly slower than other aligners for both experiments (Figure 11). ARYANA not only finished alignment the fastest, but it also left the least number of reads unaligned (Figure 12).

## Conclusion

There are many different factors that lead to the impressive performance of ARYANA. The algorithm we use to extract seeds collects a smaller set of seeds in compare to the classic approach of using fixed seeds, thus reducing the total time spent on matching them to the reference genome while not significantly losing precision and recall. Furthermore, our algorithm extracts these seeds much faster than the naive approaches that extract the same seeds. The main reason for this is that in many cases our algorithm is confident that a seed will fail to match to the reference based on the information it had gained when it was matching the previous seeds. Additionally, the data structure we have used (the hash table) to manage the information regarding the possible genomic positions of the read (tags) provides functions to update and access this genome wide information fast enough to be guaranteed of no overall time overhead, while consuming an inconsiderable amount of memory. Finally the previously matched blocks during the first phase and the approach we have in the dynamic programming algorithm have generally- decreased the time spent for the second phase.

In overall, our results on both simulated and experimental data are evident for the efficient and accurate algorithmic architecture used in ARYANA. We have developed ARYANA such that it would be convenient to use the same architecture in development of the mission-specific aligners for analysing the other types of biological data.

## Competing interests

The authors declare that they have no competing interests.

## Authors' contributions

A.Sh., M.Gh., A.A., and M.S. conceived the project. A.Sh., M.Gh., and A.A. defined the problem and designed the algorithm. A.A. and M.Gh. implemented the algorithms. A.A., M.Gh. and A.Sh. ran the experiments and interpreted the results. M.Gh., A.A., A.Sh., and H.Ch. wrote the manuscript.

## Declarations

Funding for this research was partially provided by the National Science Foundation through grant number DBI-1262565 to H.Ch.

This article has been published as part of BMC Bioinformatics Volume 15 Supplement 9, 2014: Proceedings of the Fourth Annual RECOMB Satellite Workshop on Massively Parallel Sequencing (RECOMB-Seq 2014). The full contents of the supplement are available online at http://www.biomedcentral.com/bmcbioinformatics/supplements/15/S9.
